# Better survival of renal cell carcinoma in patients with inflammatory bowel disease

**DOI:** 10.18632/oncotarget.5186

**Published:** 2015-10-05

**Authors:** Lauranne A.A.P. Derikx, Loes H.C. Nissen, Joost P.H. Drenth, Carla M. van Herpen, Wietske Kievit, Rob H.A. Verhoeven, Peter F.A. Mulders, Christina A. Hulsbergen-van de Kaa, Marye J. Boers-Sonderen, Tim R.A. van den Heuvel, Marieke Pierik, Iris D. Nagtegaal, Frank Hoentjen

**Affiliations:** ^1^ Inflammatory Bowel Disease Centre, Department of Gastroenterology and Hepatology, Radboud university medical centre, Nijmegen, The Netherlands; ^2^ Department of Medical Oncology, Radboud university medical centre, Nijmegen, The Netherlands; ^3^ Radboud Institute for Health Sciences, Radboud university medical centre, Nijmegen, The Netherlands; ^4^ Netherlands comprehensive cancer organization / Netherlands Cancer Registry; ^5^ Department of Urology, Radboud university medical centre, Nijmegen, The Netherlands; ^6^ Department of Pathology, Radboud university medical centre, Nijmegen, The Netherlands; ^7^ Department of Gastroenterology and Hepatology, Maastricht University Medical Centre, Maastricht, The Netherlands

**Keywords:** inflammatory bowel disease, renal cell carcinoma, immunosuppressive therapy

## Abstract

**Background:**

Immunosuppressive therapy may impact cancer risk in inflammatory bowel disease (IBD). Cancer specific data regarding risk and outcome are scarce and data for renal cell carcinoma (RCC) are lacking. We aimed(1) to identify risk factors for RCC development in IBD patients (2) to compare RCC characteristics, outcome and survival between IBD patients and the general population.

**Methods:**

A PALGA (Dutch Pathology Registry) search was performed to establish a case group consisting of all IBD patients with incident RCC in The Netherlands (1991–2013). Cases were compared with two separate control groups: (A) with a population-based IBD cohort for identification of risk factors (B) with a RCC cohort from the general population to compare RCC characteristics and outcomes.

**Results:**

180 IBD patients with RCC were identified. Pancolitis (OR 1.8–2.5), penetrating Crohn's disease (OR 2.8), IBD related surgery (OR 3.7–4.5), male gender (OR 3.2–5.0) and older age at IBD onset (OR 1.0–1.1) were identified as independent risk factors for RCC development. IBD patients had a significantly lower age at RCC diagnosis (*p* < 0.001), lower N-stage (*p* = 0.025), lower M-stage (*p* = 0.020) and underwent more frequently surgical treatment for RCC (*p* < 0.001) compared to the general population. This translated into a better survival (*p* = 0.026; HR 0.7) independent of immunosuppression.

**Conclusions:**

IBD patients with a complex phenotype are at increased risk to develop RCC. They are diagnosed with RCC at a younger age and at an earlier disease stage compared to the general population. This translates into a better survival independent of immunosuppressive or anti-TNFα therapy.

## INTRODUCTION

Inflammatory bowel disease (IBD), including ulcerative colitis (UC), Crohn's disease (CD) and indeterminate colitis is a chronic inflammatory disorder of the gastrointestinal tract. Patients with this disease have an increased risk for both intestinal and various extra-intestinal malignancies [1, 2]. This risk is mainly attributed to two drivers: chronic inflammation and drug-induced immunosuppression [3]. Particularly immunosuppressive medication such as thiopurines and methotrexate may play a role in the development of extra-intestinal malignancies by impairing immunosurveillance of tumor cells or inducing DNA damage [4–7]. The potential associated cancer risk is an important growing concern given the need for prolonged immunosuppressive therapy in IBD, especially in view of the aging IBD population.

Various extra-intestinal malignancies, such as lymphoproliferative disorders and non-melanoma skin cancers occur more frequently in IBD patients compared to the general population, mainly in those using immunosuppression [2, 6, 8–12]. Although only limited evidence is available, it has been suggested that immunosuppression in IBD patients may increase the risk for a variety of solid malignancies, such as renal cell carcinoma (RCC). Indeed, RCC occurs more frequently in post-transplantation patients exposed to immunosuppressive medication [13]. In addition, the risk for urinary tract cancers in IBD patients on thiopurines seems to be elevated [3].

It is unknown whether and how IBD therapy impacts risk of cancer recurrence, outcome and survival. Aggregate data failed to demonstrate an effect of immunosuppression and anti-TNFα agents on recurrence of any cancer in IBD patients [5]. By contrast, cancer specific data on recurrence and outcome are scarce. For example, only eight case reports of IBD patients with RCC have been described and led to speculation on a putative association with immunosuppressive therapy [14–19]. As such, more data are needed to estimate RCC risk and to guide the subsequent individual IBD therapy.

To this end we established a nationwide cohort of IBD patients with incident RCC. We had a dual aim: (1) to identify risk factors for RCC development, and in particular to investigate the impact of IBD therapy on RCC development (2) to compare RCC characteristics, outcome and survival between IBD patients and the general population, including the impact of immunosuppression and anti-TNFα agents.

## RESULTS

### Patient identification

**Figure 1:**
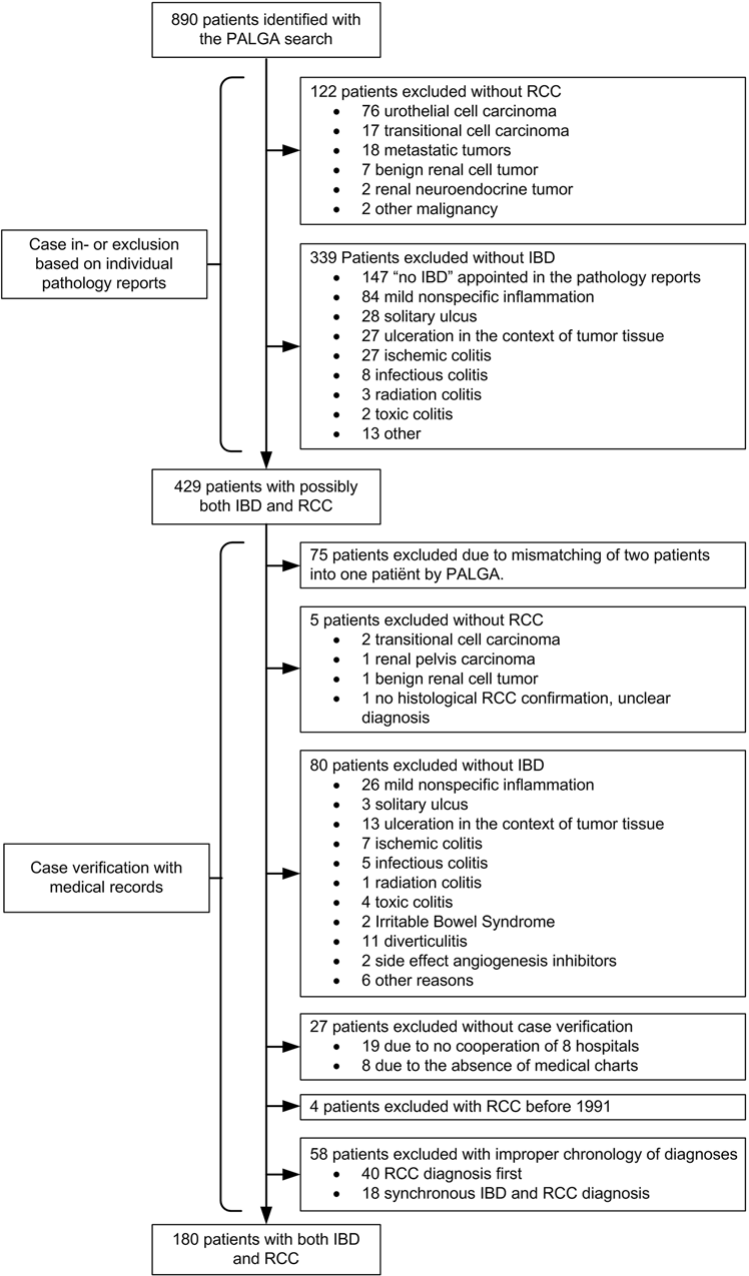
Patient inclusion flowchart IBD, inflammatory bowel disease; RCC, renal cell carcinoma

We identified 180 IBD patients who developed RCC in 69 of 87 hospital organizations in the Netherlands (Figure 1) [20]. Twenty-seven potential cases could not be verified and were excluded. To identify risk factors for RCC development we established a control group of 1800 IBD patients randomly selected from the IBD South Limburg Cohort (IBDSL; Case control study A). For the comparison of RCC characteristics and outcomes we identified a second control group using the Eindoven Cancer Registry (ECR). This search yielded 4388 patients with RCC in the general population (Case control study B).

### Risk factors for RCC development - Case control study A

Potential clinical and demographic risk factors for RCC development were compared univariable between IBD cases who developed RCC and IBDSL control patients (Table 1). Male gender, Montreal E3 pancolitis, perianal disease activity, a stricturing and/or penetrating CD phenotype, and IBD related surgery occurred statistically significantly more frequent in the case group (*p* < 0.001 for all comparisons). Furthermore, cases had a statistically significantly longer duration of follow up since IBD diagnosis (*p* < 0.001), but used less thiopurines (*p* = 0.047) and anti-TNFα agents (*p* = 0.006) during follow up. We considered differences in inclusion period (IBD diagnosis since 1950 (cases) versus IBD diagnosis since 1991 (controls)) as a reason for these differences, since widespread use of thiopurines and the introduction of anti-TNFα therapy occurred in the last decade of inclusion. Using similar inclusion periods of IBD diagnosis for both cases and controls (since 1991) almost abolished treatment differences (5-aminosalicylic acids (5-ASA), 89.6% (cases) versus 89.8% (controls), *p* = 0.954; thiopurines, 35.6% versus 40.2%, *p* = 0.432; methotrexate, 0.0% versus 5.3%, *p* = 0.049; cyclosporine, 4.1% versus 1.5%, *p* = 0.102; anti-TNFα therapy, 15.1% versus 19.7%, *p* = 0.326).

**Table 1:** Univariable comparison of potential risk factors and confounders between cases (IBD patients who developed RCC) and controls (randomly selected IBD patients from IBDSL) for the identification of risk factors to develop RCC (case control study A)

Variable	IBD and RCC cases (*n* = 180)	IBDSL (*n* = 1800)	Missing values (cases/IBDSL)	*P*-value
**Male gender**, *n* (%)	114 (63.3)	837 (46.5)	0	<0.001
**Ever smoked**^a^, *n* (%)	38 (62.3)	421 (62.5)	11/122	0.979
**Age at IBD diagnosis**(y), median	43	39	3/1	0.106
**IBD type**^b^ **Ulcerative colitis**, *n* (%) **Crohn's disease**, *n* (%)	93 (56.4) 72 (43.6)	1004 (55.8) 796 (44.2)	0	0.885
**Ulcerative colitis** **Extend** **Proctitis (E1)**, *n* (%) **Left-sided colitis (E2)**, *n* (%) **Pancolitis (E3)**, *n* (%)	14 (17.5) 24 (30.0) 42 (52.5)	243 (24.4) 472 (47.5) 279 (28.1)	10/13	<0.001
**Crohn's disease** **Extend** **Ileum (L1)**, *n* (%) **Colon (L2)**, *n* (%) **Ileocolonic (L3)**, *n* (%) **Upper digestive (L4)**, *n* (%) **Perianal disease activity**, *n* (%) **Phenotype** **Non stricturing/penetrating (B1)**, *n* (%) **Stricturing (B2**), *n* (%) **Penetrating (B3),** *n* (%) **Stricturing and penetrating**, *n* (%)	27 (38.6) 19 (27.1) 24 (34.3) 2 (2.8) 21 (30.0) 19 (27.9) 16 (23.5) 15 (22.1) 18 (26.5)	223 (28.1) 183 (23.0) 389 (48.9) 65 (8.2) 119 (14.9) 437 (54.9) 171 (21.5) 96 (12.1) 92 (11.6)	2/1 1/0 2/0 4/0	0.054 0.106 0.001 <0.001
**Medical therapy prior to RCC diagnosis** **5ASA**, *n* (%) **Thiopurines**, *n* (%) **Methotrexate**, *n* (%) **Cyclosporine**, *n* (%) **Anti-TNFα therapy**, *n* (%)	145 (94.2) 49 (32.0) 3 (2.0) 5 (3.3) 16 (10.5)	1605 (89.8) 717 (40.2) 95 (5.3) 26 (1.1) 350 (19.7)	26/13 27/17 29/10 27/10 28/25	0.083 0.047 0.074 0.091 0.006
**IBD related surgery**, *n* (%)	86 (48.0)	508 (28.3)	1/8	<0.001
**Calendar year of IBD diagnosis**, median	1989	2003	3/1	<0.001
**Duration of follow up since IBD diagnosis** (y), median	19	7	3/30	<0.001

IBD, Inflammatory bowel disease; IBDSL, IBD South Limburg cohort; RCC, renal cell carcinoma.

a Smoking data were only available for patients with Crohn's Disease

b Indeterminate colitis was not considered in this comparison since these patients were excluded from IBDSL

A multivariable logistic regression model that took the duration of follow up since IBD diagnosis into account was made separately for UC and CD patients to identify independent risk factors for RCC development. Included variables were: gender, age at IBD diagnosis, extend of UC and CD, perianal disease activity, CD phenotype and IBD related surgery. As prescribed medical therapy might be different and/or not reliable in early years of inclusion, we did not include these variables in this model. Therefore, we performed a sensitivity analysis including patients with an IBD diagnosis since 1991 in both the case and control group. Medical therapy was included in this logistic regression model.

Table 2 shows the final logistic regression models after backward elimination of the non-significant variables for both UC and CD patients. Patients with a more complex phenotype including Montreal E3 UC (OR 1.8–2.5, 95% CI 1.0–5.3), penetrating CD (OR 2.8, 95% CI 1.3–5.8) and/or IBD related surgery (OR 3.7–4.5, 95% CI 1.6–8.2) were at increased risk for RCC development. Furthermore, male gender (OR 3.2–5.0, 95% CI 1.7–13.2) and older age at IBD diagnosis but not age by itself (OR 1.0–1.1, 95% CI 1.0–1.1) were identified as independent risk factors. Use of 5-ASA (OR 0.2, 95% CI 0.0–0.7) protected against RCC development.

**Table 2:** Final multivariable regression model for the identification of independent risk factors to develop RCC

Model	Variable	Coefficient β	Odds ratio (95% confidence interval)	*P*-value
**Ulcerative colitis** (all cases, *n* = 1061)	Male gender IBD related surgery Age at IBD diagnosis Montreal E3 colitis^a^	1.169 1.499 0.023 0.598	3.218 (1.715–6.040) 4.477 (2.433–8.238) 1.023 (1.006–1.042) 1.818 (1.045–3.163)	<0.001 <0.001 0.009 0.034
**Ulcerative colitis** (sensitivity analysis, *n* = 1015)	Male gender IBD related surgery Age at IBD diagnosis Montreal E3 colitis^a^ 5-ASA	1.609 1.306 0.028 0.922 –1.746	4.999 (1.889–13.226) 3.692 (1.578–8.641) 1.029 (1.006–1.051) 2.513 (1.2005.262) 0.174 (0.044–0.687)	0.001 0.003 0.011 0.015 0.013
**Crohn's disease** (all cases, *n* = 845)	Age at IBD diagnosis Penetrating disease	0.035 1.021	1.035 (1.014–1.057) 2.776 (1.320–5.836)	0.001 0.007
**Crohn's disease** (sensitivity analysis, *n* = 811)	Age at IBD diagnosis	0.049	1.051 (1.028–1.074)	<0.001

Similar inclusion periods of IBD diagnosis (since 1991) for cases and controls were used in the sensitivity analysis (case control study A). IBD, inflammatory bowel disease; 5-ASA, 5-aminosalicylic acids.

a Reference category is patients with Montreal E1 or E2 colitis

### RCC characteristics and survival - Case control study B

Univariable comparisons of RCC characteristics between IBD cases and the general population are shown in Table 3. IBD patients had a statistically significantly lower age at RCC diagnosis (*p* < 0.001), lower N-stage (*p* = 0.025), lower M-stage (*p* = 0.020) and underwent more frequently surgical treatment for RCC (*p* < 0.001). This may be attributed to a high percentage of incidentally diagnosed cancers in the case group (*n* = 80/180, 51.3%).

**Table 3:** Univariable comparison of RCC characteristics between cases (IBD patients who developed RCC) and controls (RCC patients in the general population derived from ECR) (case control study B)

Variable	IBD and RCC cases (*n* = 180)	ECR (*n* = 4388)	Missing values(cases/ECR)	*P*-value
**Male gender**, *n* (%)	114 (63.3)	2659 (60.6)	0	0.461
**Age at RCC diagnosis** (y), median	62 (27–83)	66	0	<0.001
**Location** **Left-sided**, *n* (%) **Right-sided**, *n* (%)	89 (50.6) 87 (49.4)	2119 (48.7) 2230 (51.3)	4/39	0.631
**Grade** **1–2**, *n* (%) **3–4**, *n* (%)	88 (72.7) 33 (27.3)	1214 (69.3) 539 (30.7)	59/2635	0.442
**T stage** **T1–T2**, *n* (%) **T3–T4**, *n* (%)	130 (76.9) 39 (23.1)	2509 (70.9) 1032 (29.1)	11/847	0.089
**N stage** **N0**, *n* (%) **N+**, *n* (%)	160 (94.1) 10 (5.9)	3281 (88.6) 423 (11.4)	10/684	0.025
**M stage** **M0**, *n* (%) **M1**, *n* (%)	155 (87.1) 23 (12.9)	2962 (80.0) 742 (20.0)	2/684	0.020
**Surgery**, *n* (%)	168 (93.9)	3318 (75.6)	1/0	<0.001
**Calendar year of RCC diagnosis**, median	2003	2007	0	<0.001

RCC, renal cell carcinoma; IBD, inflammatory bowel disease; ECR, Eindhoven cancer registry.

**Figure 2:**
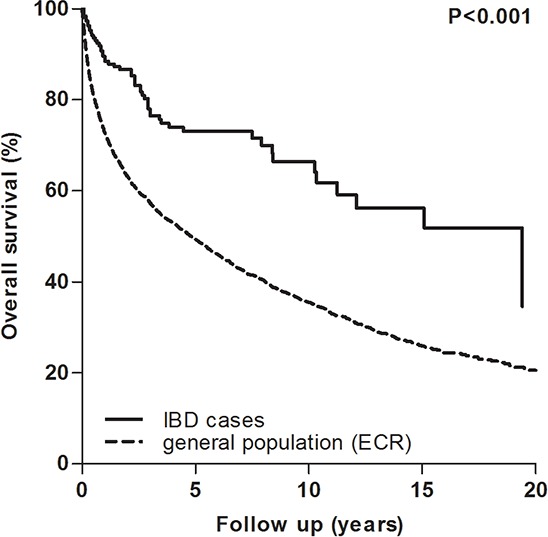
Overall survival curves of the general and IBD population following RCC diagnosis IBD, inflammatory bowel disease; ECR, Eindhoven cancer registry

Figure 2 displays the overall survival curves of the case and control group. IBD patients had a statistically significant better overall survival compared to the general population (*p* < 0.001). However, age at RCC diagnosis, T-stage, M-stage, surgical treatment and calendar year of RCC diagnosis emerged as confounders in a Cox model. Adjusted for these confounders, a protective effect of IBD on overall survival was still present (*p* = 0.026; HR 0.7; 95% CI 0.5–1.0).

### RCC survival related to medical IBD therapy

**Figure 3:**
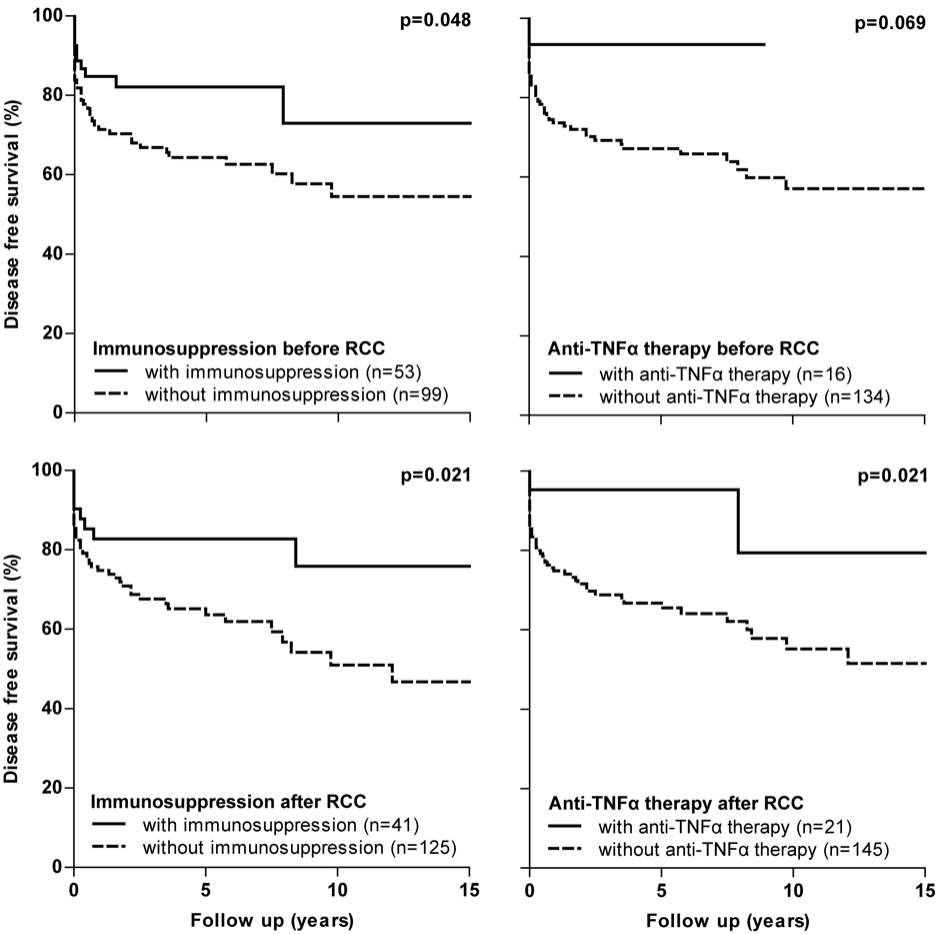
Disease free survival curves in IBD subgroups with RCC based on IBD medication received RCC, renal cell carcinoma

Based on received IBD medication, we performed subgroup survival analysis for IBD cases with RCC. Patients who used immunosuppression (including thiopurines, methotrexate and cyclosporine) and/or anti-TNFα therapy before or after RCC diagnosis had a statistically significantly better disease free survival following RCC diagnosis compared to those who did not (Figure 3). Especially patients who were treated with immunosuppression and/or anti-TNFα agents after RCC diagnosis, showed a better disease free survival. However, a multivariable Cox analyses adjusted for the confounders TNM stage and age at RCC diagnosis, abolished this protective effect of immunosuppressive and anti-TNFα therapy (immunosuppression before RCC diagnosis, *p* = 0.946; immunosuppression after RCC diagnosis, *p* = 0.386; anti-TNFα therapy before RCC diagnosis, *p* = 0.673; anti-TNFα therapy after RCC diagnosis, *p* = 0.502). Similar survival curves were found for the effect of IBD therapy on overall survival (data not shown).

Following a similar strategy as for the identification of risk factors we performed a sensitivity analysis focusing on patients who carried an IBD diagnosis since 1991. We determined the effect of medical therapy on disease free and overall survival. All survival analyses and Cox models showed similar results as shown above (data not shown).

## DISCUSSION

One of the key findings of our study is that IBD patients with a complex phenotype (including Montreal E3 UC, penetrating CD and/or IBD related surgery) are at increased risk to develop RCC. They are younger at diagnosis and carry a lower RCC stage compared to the general population. This translates into a better disease free and overall survival. The second key finding of our study is that immunosuppressive and anti-TNFα therapy does not adversely affect disease free and overall survival in IBD patients following RCC diagnosis.

A better survival in our IBD cohort with RCC may be caused by frequent abdominal imaging in these patients, which leads to incidental findings such as RCC. Due to the widespread use of imaging techniques, the incidental detection of RCC in the general population significantly increased in recent decades to approximately 40% [21–23]. This compares to 51% for incidentally detected RCC in our IBD cohort. Previous studies have shown that patients with these incidentally detected RCC are diagnosed at an earlier stage, which is translated into a better survival after correction for confounders (TNM stage, age at RCC diagnosis, calendar year) [22, 24]. This is in line with our study in which IBD patients (including a high proportion of incidentally diagnosed cancers) received earlier RCC diagnosis and had a better survival.

We found that patients with a more complex IBD phenotype are at increased risk to develop RCC. A more frequent and intensive use of the health care system, including abdominal imaging, may be associated with this phenomenon. Indeed, another study found that IBD patients exposed to anti-TNFα agents (generally prescribed for patients with a more complex IBD phenotype) developed RCC at a younger age and received earlier RCC surgery compared to IBD patients unexposed to anti-TNFα therapy or patients having rheumatoid arthritis [25]. Other risk factors for RCC development were male gender and older age at IBD onset (not age by itself), but not the use of medical therapy. One could hypothesize that with increasing age, potential cancer risk factors accumulate until IBD onset with subsequently accelerated carcinogenesis. As such, patients who develop IBD later in life are at increased risk to develop early colorectal cancer (<8 y) and more widespread colorectal neoplasia [26, 27]. The role of immunosuppression and/or anti-TNFα agents in relation to cancer development remains to be clarified as the literature reports contradictory results [3, 5, 28].

Results of our study demonstrated no adverse effect of immunosuppression and/or anti-TNFα therapy on both disease free and overall survival following RCC. These therapies were mainly (re)started or continued after RCC diagnosis in patients with low stage RCC and as a corollary these patients showed a better disease free and overall survival (Figure 3). For example, 32 out of 41 patients (82.1%) who used immunosuppressive therapy and 17 out of 21 patients (81.0%) with anti-TNFα therapy after RCC diagnosis had a T1 RCC. Adjustment for TNM stage abolished the protective effect of immunosuppressive and anti-TNFα therapy and no differences on survival were subsequently found. These findings are in line with the only available, prospective study in IBD patients, which showed no negative impact of immunosuppressive agents on recurrent cancer of any type [5]. Other data originates from observational studies including patients with rheumatoid arthritis or solid organ transplants. No difference in any new or recurrent malignancy incidence was observed in rheumatoid arthritis patients exposed or unexposed to anti-TNFα agents [7, 29]. A study in post-transplant setting demonstrated a recurrence rate of 0% for incidentally diagnosed RCC and of 30% for symptomatic RCC, although a formal comparison to a control group was lacking [30].

Despite concerns regarding a cancer inducing effect of anti-TNFα therapy, TNFα blockers have been previously considered as a therapeutic strategy for RCC [31, 32]. Previous studies showed that TNFα can act as an autocrine tumor growth factor and that its presence is associated with poor prognosis. Indeed, phase I/II trials in RCC demonstrated an anti-tumor effect of anti-TNFα treatment [32]. However, the most recent phase II trial in 2010 showed no beneficial effect of anti-TNFα therapy in metastatic RCC [31]. Similarly, results of our study did not show a better survival of metastatic RCC in patients treated with anti-TNFα agents (data not shown).

Our study has important clinical implications for the evidence-based management of IBD therapy in patients with a history of RCC. As no adverse effect of IBD therapy on disease free and overall survival was observed, our data suggest that these agents could be considered following RCC. Cancer specific data are lacking to date, although case-by-case management is encouraged based on the characteristics and expected evolution of the cancer, the probable impact of IBD therapy on cancer evolution, and IBD severity [4, 7]. The impact of dose, duration and time interval following RCC remains to be assessed in larger prospectively followed cohorts. In addition, more cancer specific data are needed for other types of cancer to develop individualized evidence-based management strategies in IBD patients with cancer.

The present study has several limitations. First, the retrospective nature of data collection could have influenced the completeness and accuracy of the extracted data. For example, medication use was difficult to ascertain from older medical records. To address this issue, we performed sensitivity analyses including patients with similar calendar years of IBD diagnosis or RCC diagnosis in the case and control group, disseminating missing values and errors across groups. Second, the use of multiple databases and registries resulted in different ways of data collection and the absence of some variables. For example, potential risk factors and confounders, such as smoking behavior, hypertension, body mass index and incidental detection of RCC, were not available in IBDSL or the ECR. Given this limitation some of our results need to be interpreted with caution. However, it was inevitable to use multiple databases to address our research questions. Finally, selection bias may have been introduced as we used different registries and databases. For example, cases were identified nationwide whereas controls with RCC and controls with IBD were ascertained from two different registries in the south of The Netherlands. However, previous studies confirmed that these population-based registries are representative of the total Dutch population [33].

In conclusion, we identified a complex IBD phenotype as a risk factor to develop RCC. IBD patients were diagnosed with RCC at a younger age and at an earlier disease stage compared to the general population, which translated into a better disease free and overall survival following RCC. Immunosuppressive and anti-TNFα therapy did not adversely affect this better survival. This observation may aid physicians in guiding IBD therapy following RCC diagnosis and treatment.

## MATERIALS AND METHODS

### Study design and data sources

In order to study risk factors and the clinical course of RCC in IBD patients, we performed two retrospective nationwide case control studies. We established a case group consisting of all IBD patients who developed RCC in The Netherlands assembled over 22 years, using PALGA (Dutch nationwide network and registry of histo- and cytopathology) [34]. Subsequently, these cases were included in the following two case control studies:

- The first case control study aimed for the identification of risk factors to develop RCC. Controls were randomly sampled from IBDSL, a population-based IBD registry [35].
- The second case control study was performed to compare RCC characteristics and outcomes between IBD patients and the general population. Controls were identified from the ECR and included patients from the general population who developed RCC [36].

The study was approved by the Privacy Commission and Scientific Council of PALGA and by the Medical Ethics Review Committee region Arnhem - Nijmegen (Registration number 2013/419).

### Case identification

PALGA was searched in order to identify all IBD patients with concomitant RCC in The Netherlands from January 1991 until May 2013. The PALGA registry contains pathology reports generated in the Netherlands since 1971 and has complete national coverage since 1991 encompassing all pathology laboratories from all academic and non-academic hospitals in the Netherlands [34]. We performed a PALGA search with the following search terms: *“ulcerative colitis”*, *“Crohn's disease”*, *“indeterminate colitis”*, or *“chronic idiopathic inflammatory bowel disease”* combined with all “*primary carcinomas of the kidney”* or *“metastasis of kidney cancer”*. Cases were further confirmed or excluded after careful evaluation of the individual pathology reports and/or medical records (Figure 1).

All patients with UC, CD or indeterminate colitis who developed a histologically confirmed RCC following IBD diagnoses were included. The diagnosis of IBD was based on a combination of clinical, endoscopic, histological and radiographic criteria [37]. The following exclusion criteria were used: no diagnosis of IBD, no diagnosis of RCC, RCC diagnosis before IBD diagnosis and RCC diagnosis before 1991.

### Controls (A)–IBD South limburg cohort

Controls for the identification of risk factors to develop RCC were randomly selected from IBDSL. IBDSL is a prospectively followed, population-based IBD registry in an area in the southeast of The Netherlands between Germany and Belgium, called South-Limburg. This area has a population of approximately 645.000 inhabitants with a low migration rate and covers one academic and two general district hospitals [38]. Adult patients in this area with a diagnosis of UC or CD based on a combination of endoscopic, radiologic and histological evidence are included in this cohort since 1991 [38]. It includes 2807 IBD patients (40.9% CD, 59.0% UC), which represents 93% of the regional IBD population [35]. We randomly included patients with an IBD diagnosis between 1991 and 2011. An unmatched study design was chosen given the relatively large number of cases, allowing adjustment for possible confounders as well as to avoid missing potential risk factors [39].

### Controls (B)–Eindhoven cancer registry

Controls to compare RCC characteristics and outcomes were identified from the ECR, maintained by the Netherlands Comprehensive Cancer Organization. The registry prospectively collects data on all newly diagnosed cancers in the southern part of The Netherlands since 1955 [36]. This area includes 10 general hospitals and 6 regional pathology laboratories, comprising approximately 2.3 million inhabitants (15% of the Dutch population) [40]. Tumor characteristics and patient characteristics are routinely extracted from medical records by specially trained administrators of the cancer registry. By means of an independent case ascertainment method, the completeness of the registration is estimated to exceed 95%. [41] We included all patients with a histologically confirmed RCC between 1991 and 2010 from the ECR as controls.

### Data extraction

Two authors (L.D and L.N) extracted demographic and clinical variables from anonymized medical records for patients included in the case group. Extracted data included gender, date of birth, smoking history (ever/never), primary sclerosing cholangitis, IBD characteristics and RCC characteristics. We collected the following IBD characteristics: type of IBD, date of diagnosis, phenotype according to the Montreal Classification, and medical and surgical treatment. Exposure to 5-ASA, thiopurines, anti-TNFα agents, cyclosporine or methotrexate was defined as “used” or “not used” since dosage/duration could not be reliably retrieved for all cases. RCC characteristics included: date of diagnosis, location, tumor stage according to the TNM classification (7^th^ edition), differentiation grade according to Fuhrman [42], whether the tumor was incidentally detected or not, treatment, outcome and survival.

Incidentally diagnosed cancers were considered to be tumors discovered during investigations performed for reasons other than for RCC related symptoms including palpable tumor, haematuria (both macroscopic and microscopic), flank pain and signs of cachexia related to the disease [21]. RCC outcome included disease free survival (duration since RCC diagnosis until recurrence or death) and overall survival (duration since RCC diagnosis until death). Histopathological subtype could not be obtained reliably due to the great variability of morphology reporting standards since 1991.

Similar variables with corresponding definitions were extracted from registries (IBDSL and ECR) for patients included in the control groups.

### Statistics

For both case control studies we compared potential risk factors, RCC characteristics and/or outcomes between cases and controls with a univariable analysis. X^2^ test or Fisher exact test (if expected cell counts were < 5) were used for categorical data and independent Student *t* test (if normally distributed) or Mann-Whitney *U* test (if not normally distributed) were used for continuous data. Variables with a *P*-value of < 0.1 in univariable analyses were included in a multivariable model. As the control group in a case control study should reflect the entire source population that gave rise to the cases, we did not exclude IBD patients who developed RCC from the control groups [43]. These patients were in both models analyzed as cases.

For case control study A, identifying independent risk factors to develop RCC, we performed a multivariable logistic regression model with backward sampling. This model was adjusted for the duration of follow up (fixed variable), which was defined as the time since IBD onset until the date of RCC diagnosis (cases) or the end of follow up or death (controls). For case control study B, comparing RCC outcome between IBD cases and the general population, we made Kaplan Meier survival curves and performed log rank analysis. Subsequently confounder correction was performed with Cox regression model with forward sampling. A covariate was considered as a confounder when the beta coefficient of the variable of interest (IBD yes/no) changed by 10% or more [44].

A *P*-value of < 0.05 (2 sided) was considered to be statistically significant. All missing values were considered to be at random and were excluded from analyses. All statistical analyses were performed with IBM SPSS statistics version 20.0 (SPSS Inc, Chicago, IL).
